# Effect of low-dose lanthanum carbonate on calcium and phosphorus metabolism in Asian Patients with end-stage renal disease, maintenance hemodialysis and hyperphosphatemia

**DOI:** 10.4314/ahs.v22i2.41

**Published:** 2022-06

**Authors:** Xiaofei Cui, Shuning Jiang, Liang Liu, Xuejie Tang, Yan Chen

**Affiliations:** 1 Emergency Intensive Care Unit, the Second Affiliated Hospital of Dalian Medical University, Dalian 116027, Liaoning, China; 2 Kidney Department, the Second Affiliated Hospital of Dalian Medical University, Dalian 116027, Liaoning, China; 3 Department of Emergency, the Second Affiliated Hospital of Dalian Medical University, Dalian 116027, Liaoning, China

**Keywords:** Low dose, Lanthanum carbonate, Hyperphosphatemia, End-stage renal disease, Hemodialysis, Asian

## Abstract

**Objective:**

This study aimed to examine whether a 12-week small-dose lanthanum carbonate (LaCO3; 500 mg/d) treatment could improve calcium and phosphorus metabolism and parathyroid function in Asian patients with end-stage renal disease (ESRD) under hemodialysis.

**Methods:**

This was a prospective observational study of patients treated at our Hospital between 10/2014 and 02/2015. The patients were given 500 mg/d of LaCO3 with lunch for 12 weeks.

**Results:**

Baseline and after 12-week treatment serum phosphorus levels were 2.49±0.51 mmol/L and 1.65±0.34 mmol/L (P<0.001). The baseline and after 12-week treatment calcium×phosphorus product were 69.40±17.34 mg2/dL2 and 44.27±9.67 mg2/dL2 (P<0.001). There was no significant difference in serum calcium and iPTH levels from baseline to after 12 weeks treatment (both P>0.05). Fourteen (25.9%) patients developed gastrointestinal adverse reactions to LaCO3 and 10 patients improved after treatment.

**Conclusion:**

Far below the 1.5–3.0g/d required by the drug instructions, LaCO3 500 mg/d for 12 weeks can still reduce serum phosphorus level and calcium × phosphorus product, without serum calcium and iPTH levels increase.

## Introduction

Phosphorus in the body is excreted mainly by the kidneys. Therefore, patients with chronic kidney disease (CKD) are prone to hyperphosphatemia, with a prevalence as high as 80% in patients under dialysis^1^. Elevated serum phosphorus may cause secondary hyperparathyroidism, disorders of vitamin D metabolism, abnormal metabolism of calcium and phosphorus, renal osteodystrophy, and coronary artery and peripheral vascular calcification^2^, thus increasing cardiovascular morbidity and mortality in patients with end-stage renal disease (ESRD)^3^, ^4^.n addition, studies showed that hyperphosphatemia is an independent risk factor for low quality of life and high financial burden in patients with ESRD^5^, ^6^.

At present, the main approaches to reduce serum phosphorus levels include hemodialysis, low phosphorus diet, phosphate binder, and surgical intervention^1^, ^7^,^8^. Because of the limitations of hemodialysis and low phosphorus diet, and because of the surgical trauma, phosphorus binder is often the preferred method for the treatment of hyperphosphatemia^1^, ^7^, ^8^. There are three types of clinical phosphate binders: aluminum, calcium, and calcium- and aluminum-free phosphate binders^9^. Aluminum is no longer recommended because of its toxicity^9^. Although the price of calcium salt is low, its long-term use may cause hypercalcemia, suppression of parathyroid gland, low-turnover bone disease, vascular and skeletal calcification, and other complications^8^, ^9^. Therefore, calcium- and aluminum-free phosphat^9^.

Among calcium- and aluminum-free phosphate binders, lanthanum carbonate is an ideal phosphate binder because of its strong phosphorus binding ability, no accumulation in human body, and low toxicity. It is more potent than calcium phosphate binders and as potent as aluminum-based ones^9^, ^10^ and can diminish the pill burden^11^. The efficacy of lanthanum carbonate decrease the serum phosphorus level has been shown by a number of clinical trials^12^–^17^. Among the current studies, there are two main ways of administration of lanthanum carbonate, one is titration first then maintain dosing and the other is oral chewing. According to the drug instruction of lanthanum carbonate, the recommended way of administration of lanthanum carbonate is oral chewing, and the recommended minimum effective dose is 750 mg/day. And so far, the reported minimum dose for oral in studies is still 750mg/day.

Nevertheless, due to economic reason, lanthanum carbonate has not been widely used in China. Therefore, the purpose of this study was to examine whether a small dose of lanthanum carbonate (500 mg/d) for 12 weeks can improve calcium and phosphorus metabolism and parathyroid function in patients with ESRD on hemodialysis.

## Methods

The study was approved by the Ethics Committees of our hospital, and all participants provided written informed consent.

### Study design and patients

This was a prospective observational study of patients with hyperphosphatemia and ESRD under maintenance hemodialysis treated at our Hospital between October 2014 and February 2015. Hyperphosphatemia was defined as serum phosphorus levels >1.78 mmol/L despite controlling phosphorus intake diet and increased frequency and duration of dialysis to at least three sessions/week and 4 h/session. The inclusion criteria were: 1) plan to initiate low-dose lanthanum carbonate treatment; 2) provided informed consent; 3) did not take any phosphate binder for 2 weeks before lanthanum carbonate treatment; 4) on dialysis for at least 3 months; and 5) >18 years of age. The exclusion criteria were: 1) hyperphosphatemia caused by factors other than ESRD; or contraindication to lanthanum carbonate.

### Treatment

Hemodialysis was performed routinely during the 12-week trial. The dialyzer was a high throughput dialyzer (FX100, Fresenius Medical Care, Hesse, Germany). The dialysate contained Ca2+ at 1.5 mmol/L. The patients were given 500 mg/d of lanthanum carbonate (Fosrenol, 500mg/tablet) to chew with lunch every day for 12 weeks. The half-life of lanthanum carbonate powder and tablets is 21.9 and 22.3 hours, respectively. Even if there are individual differences, the half-life of lanthanum carbonate is still within the range of 16.2–28.3 hours^1^.

### Biochemistry

The levels of serum phosphorus, serum calcium, and intact parathyroid hormone (iPTH) were detected as baseline and after 4, 8, and 12 weeks of treatment. In the early morning before dialysis, fasting blood samples were collected, and the examinations was performed by the hospital laboratory. Serum calcium, phosphorus, and iPTH levels were measured using commercial kits (Roche Diagnostics, Basel, Switzerland) on a DXI800 fully automatic chemiluminescence immunoassay analyzer (Beckman Coulter, Brea, CA, USA). The normal ranges of calcium, phosphorus, and iPTH levels were 2.23–2.53 mmol/L, 0.81–1.45 mmol/L, and 10–65 pg/mL^18^.

### Data collection

Monitor the patients' liver function and check if patients have headache, nausea, vomiting, abdominal pain and other adverse reactions at each dialysis session. Demographic and clinical data were extracted from the medical charts.

### Statistical analysis

The distribution of the continuous variables was assessed using the Kolmogorov-Smirnov test. Normally distributed continuous data were presented as mean ± standard deviation and analyzed using repeated measure ANOVA with the Dunnett's T3 test. Categorical data were presented as frequencies and analyzed using the McNemar test. All analyses were performed using SPSS 19.0 (IBM, Armonk, NY, USA). Two-sided P-values <0.05 were considered statistically significant.

## Results

### Characteristics of the patients

Sixty consecutive patients were evaluated for inclusion. Among them, six patients were with hyperphosphatemia caused by factors other than ESRD or contraindications to lanthanum carbonate. Thus, 54 patients were selected. Among them, 16 patients withdrew from the study because of adverse reactions, economic reasons, death, transfer to another hospital, and other reasons. Therefore, complete data were available for 38 patients over 12 weeks.

Table 1 presents the characteristics of the patients. These 38 patients included 22 males and 16 females, with an average age of 55.6±15.7 years. Duration of dialysis was 26.2±28.9 months. All patients received three dialysis sessions/week for 4 h/session.

**Table 1:** Characteristics of the patients

	Completed the 12- week treatment	Did not complete the 12- week treatment
	n=38	n=16
Age (years)	55.6±15.7	58.4±12.3
Male, n (%)	11 (29.0)	9 (56.3)
Dialysis duration (months)	26.2±28.9	29.4±15.4
Dialysis frequency (/week)	3.0±0.0	3.0±0.0
Dialysis duration (hours)	4.0±0.0	4.0±0.0
Primary disease Chronic glomerulonephritis	24 (63.2%)	12(75.0%)
Diabetic nephropathy	4 (10.5%)	1 (6.3%)
Goodpasture syndrome	2 (5.3%)	0
Kidney damage by drugs	2 (5.3%)	0
Unknown	6 (15.8%)	3 (18.8%)

### Serum calcium, phosphorus, calcium phosphorus product, and iPTH level

In the 38 patients in this study, the serum phosphorus level before treatment was (2.49 ± 0.51) mmol / L, and after 12 weeks of treatment, the serum phosphorus level was (1.65 ± 0.34) mmol / L, and the overall average serum phosphorus level decreased by 0.84 from baseline mmol / L (P <0.001), with statistical significance. The calcium and phosphorus product was (69.40 ± 17.34) mg^2^ / dl^2^ after 12 weeks treatment decreased to (44.27 ± 9.67) mg^2^ / dl^2^, which was 21.13 mg^2^ / dl^2^ (P <0.001) lower than the baseline value, with statistical significance. Serum calcium level and serum iPTH level showed no significant difference compared with before and after treatment (both P> 0.05).

### Adverse events

Among the initial 54 patients (Table 3), 14 patients developed adverse reaction to lanthanum carbonate, which were all gastrointestinal adverse reactions. Constipation occurred in 12 patients; 10 of them improved after symptomatic treatments and two withdrew from the study because symptoms could not improve. Nausea occurred in four patients; after symptomatic treatment, they improved and continued to participate in the study. Bowel habits changed in two patients, and they withdrew from the study. In this study, no adverse reaction of lanthanum carbonate was observed on the nervous, respiratory, or circulatory system. Death occurred in two patients during treatment. The causes of death were acute cerebral infarction and myocardial infarction, respectively.

**Table 3:** Adverse reactions of lanthanum carbonate

	n	%	Improved after treatment (n)	Quit (n)
Constipation	12	22.2%	10	2
Bowel habit changes	2	3.7%	0	2
Nausea	4	7.4%	4	0

The adverse events were recorder for the original 54 patients.

## Discussion

Lanthanum carbonate decreases serum phosphorus levels, but almost all studies examined doses of 750–3000 mg/day, which is expensive. Therefore, this study aimed to examine whether a small dose of lanthanum carbonate (500 mg/d) for 12 weeks could improve the calcium and phosphorus metabolism and parathyroid function in patients with ESRD under hemodialysis. The results showed that small doses of lanthanum carbonate (500 mg/d) for 12 weeks can reduce serum phosphorus levels and calcium phosphorus product, without increase in serum calcium and serum PTH levels. Adverse effects were mostly mild and manageable. Serum phosphorus and calcium phosphorus product are the main indicators of hyperphosphatemia^1^, ^6^, ^8^. Indeed, the K/DOQI guidelines points out that serum phosphorus level of patient with stage CKD5 should be controlled to 1.13–1.78 mmol/L, serum calcium level to 2.1–2.42 mmol/L, and calcium phosphorus product to <55 mg^2^/dL^2^ 19. Therefore, based on those guidelines, the present study aimed to reduce serum phosphorus level to <1.78 mmol/L and calcium phosphorus product to <55 mg^2^/dL^2^. Among the 38 patients in this study, serum phosphorus levels decreased in 30 patients within 12 weeks; among whom, serum phosphorus levels of 26 (68%) patients decreased to <1.78 mmol/L. These results are supported by previous studies^14^–^17^,^20^–^22^. Importantly, those previous studies used lanthanum carbonate doses of 750–3000 mg/day, but this study still achieved a control rate of serum phosphorus level comparable to them. Of course, this comparison is purely anecdotic and needs more studies including low and higher doses. Compared with those previous studies^15^–^17^, ^20^–^22^, this study shows that a dose of 500 mg/d of lanthanum carbonate, despite being significantly lower, was still efficient after 12 weeks.

Compared with traditional calcium phosphate binders, the major advantage of lanthanum carbonate is that it does not increase serum calcium level while decreasing serum phosphorus level, and it could decrease the risk of vascular calcification. A meta-analysis of 11 clinical trials with a total of 1501 patients with CKD showed that compared with calcium phosphate binders, lanthanum carbonate can avoid the occurrence of hypercalcemia^23^. Another meta-analysis of 18 clinical trials with a total of 3676 patients with CKD also showed that compared with calcium phosphate binders, lanthanum carbonate can effectively delay the process of cardiovascular calcification^24^. In this study, after taking 500 mg/day lanthanum carbonate for 12 weeks, the serum calcium level did not increase, but had a decreasing trend with no statistical significance. In the future, the sample size could be expanded and the observation time could be prolonged to explore whether the serum calcium level is indeed decreasing. Also suggesting that in the long-term of using lanthanum carbonate should monitor serum calcium level, and if necessary replenish calcium for the patient in time.

At present, the effects of lanthanum carbonate on serum iPTH level differ among countries and populations. A number of factors could be involved in these discrepancies, including characteristics of the patients, genetics, study design, sample size, and magnitude of changes in biochemical parameters. A self-controlled study by Ishizu et al.^25^ showed that after 40 hemodialysis patients continuously took lanthanum carbonate for 24 months, there was no significant difference in serum iPTH levels before and after treatment. In a study by Hutchison et al.^7^, the dose of lanthanum carbonate was 375–2250 mg/day and serum iPTH levels decreased after 4 weeks. Sprague et al.^26^, Shigematsu et al.^15^, and Huang et al.^27^ showed that there was no significant difference between lanthanum carbonate and placebo for iPTH levels. on the other hand, Chiang et al.^28^, and Finn et al.^29^ showed that lanthanum carbonate can decrease serum iPTH levels. Compared with calcium phosphate binders, Zhai et al.[30 showed, using nine studies, that the decrease of serum iPTH level by lanthanum carbonate was higher than that by calcium phosphate binders. Among the 38 patients in this study, there was no significant difference in serum iPTH level before and after treatment with lanthanum carbonate. As the serum calcium level of patients has a tendency to decrease after treatment with no statistical significance, it is hardly to speculate whether the serum iPTH level was affected. Therefore, the effect of lanthanum carbonate on the serum iPTH level of patients remains to be further researched by increasing the sample size and extending the research time.

Lanthanum carbonate is mainly dissociated in the acidic environment of the digestive tract, and it is especially suitable for patients with chronic kidney disease because it is not excreted via kidneys^8^, ^17^, ^30^. In this study, no significant adverse reactions of lanthanum carbonate on the nervous system were observed, and liver function tests showed no obvious change. Dellanna et al.^21^ found that the drug-related adverse reaction of lanthanum carbonate was only 2%. At present, the most common adverse reactions included nausea, vomiting, abdominal pain, dialysis-related hypotension, muscle twitching, and myalgia^31^. Among the initial observation of 54 patients in the present study, adverse reactions to lanthanum carbonate occurred in 18 patients, and all of them were digestive system manifestations. Recent reports showed that lanthanum carbonate deposits in the stomach and duodenum mucosa^32^–^36^.The combination of lanthanum carbonate with phosphorus in food forms a lanthanum phosphate complex that is not absorbed by the digestive tract, thereby inhibiting the absorption of phosphate and reducing the levels of phosphate and calcium phosphate in the body.

Of course, the present study is not without limitations. Indeed, this research was only conducted on Asians, lacking of the research on other races. The sample size was small and from a single center. Self-control and only one dose was used. Finally, the follow-up was short. Additional studies are necessary to determine whether low-dose lanthanum carbonate is effective in controlling serum phosphorus levels in patients with ESRD and dialysis.

## Conclusion

This study showed that a small dose of lanthanum carbonate (500 mg/d) for 12 weeks could effectively lower serum phosphorus levels, without affecting serum calcium and iPTH levels in Asian patients. Further studies are needed for the long-term effects this small dose of lanthanum carbonate on calcium and phosphorus metabolism in other races patients with hyperphosphatemia, ESRD, and maintenance hemodialysis.

## Figures and Tables

**Table 2:** Changes of serum calcium, phosphorus, calcium phosphorus product, and iPTH of patients at baseline and 4, 8, and 12 weeks of lanthanum carbonate treatment (500 mg/d)

	Baseline	4 weeks	8 weeks	12 weeks
Serum calcium (2.10–2.55mmol/L)	2.15±0.18	2.14±0.13	2.06±0.16	2.05±0.05
Serum phosphorus (0.9–1.6mmol/L)	2.49±0.51	2.29±0.46*	1.87±0.36**	1.65±0.34**
Calcium phosphorus product (23.44–50.59mg^2^/dL^2^)	69.40±17.34	60.67±14.84*	50.40±8.91**	44.27±9.67**
iPTH (12–88pg/ml)	185.58±109.48	184.83±118.23	174.84±123.66	164.73±96.20

* P<0.05

** P<0.01 vs. baseline. n=38
